# Dependence of the Heterosis Effect on Genetic Distance, Determined using Various Molecular Markers

**DOI:** 10.1515/biol-2020-0001

**Published:** 2020-02-28

**Authors:** Agnieszka Tomkowiak, Jan Bocianowski, Michał Kwiatek, Przemysław Łukasz Kowalczewski

**Affiliations:** 1 Department of Genetics and Plant Breeding, Poznań University of Life Sciences, 11 Dojazd Street, 60-632 Poznań, Poland; 2 Department of Mathematical and Statistical Methods, Poznań University of Life Sciences, 28 Wojska Polskiego Street, 60-637 Poznań, Poland; 3 Institute of Food Technology of Plant Origin, Poznań University of Life Sciences, 31 Wojska Polskiego Street, 60-624 Poznań, Poland

**Keywords:** degree of relatedness, genetic similarity, heterosis, molecular markers

## Abstract

A number of studies have shown that the greater the genetic diversity of parental lines, the greater the heterosis effect. Genetic or phenotypic variation can be estimated by genotype testing on the basis of the observations obtained through prediction (a priori) or the observations and studies (a posteriori). The first method uses data such as the genealogy of a given subject and the information about its geographical origin. The second method is based on the phenotypic observation and studies, as well as on the molecular research. The development of molecular genetics and genotype testing methods at the DNA level has made it possible to rapidly assess the genetic variability regardless of the modifying effect of the environment. The aim of this study was to determine the relationship between the degree of relatedness and the DNA polymorphism (determined using AFLP, RAPD, and SSR markers) of inbred maize lines and the effect of hybrid-form heterosis. Our analysis demonstrated that the parental components for heterosis crosses can be selected on the basis of the genetic similarity determined using the molecular SSR markers and the Jaccard, Kluczyński, Nei, and Rogers coefficients. Molecular AFLP markers proved less useful for selecting the parental components, but may be used to group lines with incomplete origin data. In the case of the RAPD markers, no clear relationship between genetic distance and the heterosis effect was found in this study.

## 1 Introduction

Maize is at present the most important cereal plant in the world. The rapid increase in the global production of maize grain is the result of its versatility, and, hence, high demand. Maize kernels can be used for direct consumption or processed into food products and are also a source of starch and oil. Grain is increasingly used for the production of biofuels. High-quality hybrid varieties play a key role in maize farming. Notably, breeding of hybrid varieties often involves the heterosis effect, which generates tangible economic benefits. Currently, maize heterosis breeding is assisted by genomic selection [1, 2, 3].

Heterosis is a genetic term that describes the beneficial effects of crossbreeding—the vigor of the first generation of hybrids. This phenomenon cannot be preserved across generations and, as the definition implies, occurs only in the first hybrid generation, in which the value of a given feature exceeds that in the best parent. To date, the phenomenon of heterosis has not been fully explained, because its symptoms are too complex to be described by one simple stipulation [4]. According to Song and Messing [5], heterosis may be a consequence of differences in the structure of the genome, especially in the distribution and presence of certain genes from a given gene family in the crossed inbred lines. By isolating a specific region of the genome of two inbred maize lines, which were subsequently sequenced and mapped, they found that the size of this region and the presence of genes from a given gene family were significantly different. Genes present in one line were absent from the other, although phenotypic signs of expression were visible in the latter. This indicated that genes from the same gene family, producing similar phenotypic effects, were located in different parts of the genome in each of the tested lines. The selection of parental forms from the available gene pool of existing varieties is one of the first stages of heterosis breeding. To date, costly and laborious methods based on multiple crossbreeding and phenotypic selection, or on the analysis of isoenzymatic profiles, have been required to properly assess genetic resources in terms of productivity, quality parameters, and susceptibility to biotic and abiotic stressors [6]. The often long and complex breeding process can be significantly shortened through selection using the DNA markers. For this purpose, the existence of a close linkage disequilibrium between the marker and the locus responsible for inheritance of the functional trait is used. This method is often referred to as marker-assisted selection (MAS). Melchinger et al. [7] derived a quantitative genetic formula for heterosis, which can consequently be explained by dominance, overdominance, and epistatic interactions between nonallelic genes. Moreover, on the basis of their findings, they defined the augmented dominance effect as the dominance effect at each locus minus half the sum of the additive × additive epistasis with all other loci. Molecular-marker-aided genetic analysis has provided a new tool for studying the genetic basis of heterosis in more detail. Studies of quantitative trait loci (QTL) in maize have shown great power in mapping loci that contribute to heterosis [8]. Hua et al. [9] and Reif et al. [10] have also contributed similar studies.

Many heterosis QTL studies have focused on yield-related traits in biparental populations [11]. Other researchers have attempted to identify a QTL for general or specific combining ability in hybrids using multiparental populations [12, 13]. Li et al. [14] conducted QTL analysis, showing that the main cause of heterosis in maize is overdominance. Extensive transcriptome profiling comparing inbred lines and their hybrids by means of DNA micro-array technology in maize [15, 16, 17] and allelic transcription variation due to *cis*-regulatory elements in maize [18] indicated that transcriptional regulation and transcriptional overdominance could play an important role as molecular mechanisms establishing hybrid vigor. Tomkowiak et al. [19] examined inbred maize lines and consequently identified three SilicoDArT markers, 4591115, 7059939, and 5587991, related to the size of crop structure features. Tomkowiak et al. [20] also tested SNP and SilicoDArT molecular markers on the same pool of plant materials in the context of selecting the parental components for heterosis crossing, and concluded that such markers could be useful in selecting plant material. Coors et al. [21] stated that predicting the effect of heterosis between groups of germplasm showing genetic similarity was not possible on the basis of the genetic distance determined using the DNA markers, but should be determined in the field experiments. Based on the experimental data, they showed that dividing germplasm into different gene pools was beneficial for making an optimal use of the heterosis. Fu [22] identified the genes that control the splitting of useful hybrid traits and conducted molecular analysis for some crop species and showed that the detection of genetic relationship between lines was sometimes insufficient when creating hybrid varieties.

The aim of the present study was to examine the relationship between the heterosis effect of hybrid and genetic similarity between parental components in maize. Three different types of molecular markers were used in this study: SSR, AFLP, and RAPD. Genetic similarity for the three marker sets was calculated using five coefficients: Jaccard [23], Kulczyński [24], Nei [25], Rogers [26] and Sokal and Michener [27]. The best methods for predicting the effects of heterosis were selected on the basis of the correlation of genetic similarity between the parental components and the heterosis effect in the hybrid forms.

## 2 Materials and Methods

### 2.1 Plant Material

The plant material used in this study consisted of thirteen hybrids and nineteen inbred lines of maize; the inbred lines were the parental components of the hybrids. The lines included both flint and dent kernel forms. The lines with flint-type kernels came from INRA in France (the Lacaune population) and from Canada (from the line Inra258 and the Canadian Gene Pool group), whereas those with dent-type kernels have their origins in different groups from the United States—namely Iowa Stiff Stalk Synthetic (BSSS), Iowa Dent (ID), and Lancaster. The maize lines and hybrids belong to the IHAR group’s plant breeding collection at Smolice, Poland (Table 1).

**Table 1:** Genetic similarity between parental components of F_1_ hybrids estimated on the basis of 528 AFLP, 234 RAPD and 262 SSR markers using various coefficients

Maternal	Paternal	Hybrids	Degree of relatedness of parental forms (%)	Selected measures of genetic similarity estimated on the basis of SSR, AFLP and RAPD molecular markers														
		
lines	lines	F1		Jaccard [23]			Kulczyński [24]			Nei [25]			Rogers [26]			Sokal and Michener [27]		
				
				AFLP	RAPD	SSR	AFLP	RAPD	SSR	AFLP	RAPD	SSR	AFLP	RAPD	SSR	AFLP	RAPD	SSR
S160	S336A	M Prosna	3	0.057	0.059	0.029	0.109	0.113	0.058	0.108	0.112	0.057	0.989	0.983	0.987	0.874	0.820	0.859
S41336	S41324A-2	O Glejt	50	0.025	0.058	0.026	0.049	0.112	0.052	0.049	0.110	0.051	0.996	0.975	0.987	0.851	0.816	0.842
S78510	S80660A	Budrys	6	0.024	0.031	0.033	0.048	0.061	0.065	0.048	0.061	0.065	0.985	0.979	0.987	0.847	0.824	0.876
S54555	S79757	Popis	0	0.075	0.055	0	0.140	0.104	0	0.140	0.103	0	0.989	0.992	0.987	0.859	0.803	0.859
S245	S41789	M Glejt	13	0.061	0.046	0.095	0.114	0.90	0.177	0.114	0.087	0.174	0.996	0.970	0.987	0.882	0.841	0.919
S311	Co255	M Wilga	50	0.067	0.087	0.029	0.125	0.161	0.056	0.125	0.161	0.056	1	0.996	0.992	0.893	0.822	0.855
S64417	S61328	Narew	4	0.029	0.168	0	0.056	0.290	0	0.056	0.288	0	0.992	0.985	0.953	0.870	0.841	0.850
S41796	S41324A-2	Blask	50	0.103	0.051	0.069	0.186	0.101	0.133	0.186	0.097	0.129	0.996	0.964	0.979	0.866	0.824	0.885
S41789	S41324A-2	Grom	50	0.114	0.031	0	0.206	0.064	0	0.205	0.061	0	0.989	0.956	0.966	0.822	0.824	0.880
S56125A	S41324A-2	Brda	0	0.147	0.082	0	0.258	0.155	0	0.256	0.151	0	0.989	0.970	0.987	0.889	0.830	0.859
S63322-3	S61328	Kozak	0	0	0.061	0	0	0.119	0	0	0.114	0	0.981	0.960	0.936	0.851	0.824	0.868
S64423-2	S61328	Bejm	8	0	0.069	0	0	0.133	0	0	0.130	0	0.981	0.966	0.953	0.851	0.822	0.850
S68911	S61328	Smok	5	0.06	0.070	0.029	0.112	0.135	0.060	0.111	0.131	0.056	0.992	0.964	0.957	0.878	0.824	0.855
Correlation between relatedness and genetic similarity (Pearson)				0.253	-0.224	0.272	0.260	-0.132	0.277	0.263	-0.221	0.278	0.613*	-0.084	0.291	-0.202	-0.113	0.074
Correlation between relatedness and genetic similarity (Spearman)				0.123	-0.316	0.449*	0.123	-0.154	0.414*	0.123	-0.316	0.419*	0.580*	-0.142	0.205	-0.138	-0.065	0.064

### 2.2 Methods

The results from the field experiments were also used in Tomkowiak et al. [38], where the effects of heterosis were correlated with other molecular markers (SilicoDArt and SNP). The two-year field experiment was conducted at two breeding stations owned by the IHAR group, at Smolice (51°42’20.813’’N, 17°9’57.405’’E) and Łagiewniki (50°47’27’’N, 16°50’40’’E) in Poland. The locations differed in soil type. The plant material was sown in 2012 and 2013 on 10-m^2^ plots in triplicate. One maize cob was selected from each replicate from ten plants for biometric measurements. In total, 3840 cobs were analyzed for the two years (10 cobs × 3 replications × 32 plants × 2 locations × 2 years = 3840). Biometric measurements were carried out in the first half of November each year and included cob length (LC), cob diameter (DC), core length (LCO), core diameter (DCO), number of rows of kernels (NRK), number of kernels per row (NKR), mass of kernels from the cob (MKC), weight of one thousand kernels (WTK), and yield.

### 2.3 Degree of Relatedness

On the basis of complete information on the origin of all ancestors of the parent lines, their degree of relatedness was calculated. For this purpose, the method proposed by Henderson [41] was used. Degree of relatedness ranged from 0 (lack of relatedness) to 100 (full relatedess).

### 2.4 Heterosis

The heterosis effects for hybrids for particular traits (LC, DC, LCO, DCO, NRK, NKR, MKC, WTK, and yield) were estimated and tested by comparing a particular hybrid with the trait mean for both parents. A table of the size of the heterosis effects for individual features of hybrid crop structure has been published in Tomkowiak et al. [20].

### 2.5 DNA Isolation

The material for molecular analysis was collected from ten-day-old seedlings obtained from grains germinated under laboratory conditions. A leaf fragment for isolation was taken from ten randomly selected plants from each line and hybrid. DNA isolation was carried out using the Genomic Mini AX PLANT DNA isolation kit ( A&A Biotechnology, Gdynia, Poland) in line with the manufacturer’s protocol. DNA concentrations were determined using a DeNovix spectrophotometer (DeNovix, Wilmington, DE, USA). Samples were diluted with Tris buffer (10 mM, pH 8.0) to a final concentration of 50 ng/μL. After isolation, the gDNA was stored at -20°C.

### 2.6 Analyses using the Simple Sequence Repeat Molecular Markers

The polymerase chain reaction (PCR) analysis included nineteen inbred lines and was performed for three biological replicates. PCR was carried out in a mixture consisting of: 5 μL water, 6.25 μL DreamTaq Green PCR Master Mix, two primers added in quantity 0.25 μL each (final concentration of primers was 20 μM), and 1 μL gDNA. The total volume of the reaction mixture was 12.75 μL per sample. The markers proposed by Smith et al. [28] were used for the analysis. Forty specific markers were used to determine the degree of polymorphism between hybrids and their parental components. After optimization, the PCR reaction was carried out in a TProfessional Basic thermocycler (Polygen, Gliwice, Poland) under the same conditions, regardless of the identified marker. The profiles differed only in primer annealing temperature, which was determined according to the primer’s melting temperature. The PCR conditions were: initial denaturation for 3 min at 94°C, 40 cycles of denaturation for 30 s at 94°C, with primer annealing for 1 minute at 54°C, 58°C, or 63°C, depending on the primer, synthesis for 1 minute at 72°C, and final extension for 5 minutes at 72°C, then stored for no more than 24 h at 4°C. Electrophoresis was carried out on a 2.5% agarose gel with Midori Green Advance DNA Stain immersed in 1 × TBE buffer. To visualize the results, a Molecular Imager Gel Doc XR transilluminator and ImageLab Software (Bio-Rad Laboratories, Watford, England) were used.

### 2.7 Identification of Amplified Fragment Length Polymorphism Molecular Markers

The amplified fragment length polymorphism (AFLP) analysis included nineteen inbred lines and were performed for three biological replicates. Three sets from Invitrogen were employed: the AFLP Analysis System I (cat. no. 10544-013), the AFLP Starter Primer Kit (cat. no. 10483-014), and the AFLP Pre-amp Primer Mix (cat. no. 10792-018). The analysis was carried out in accordance with the instructions provided with the sets. In the first stage, genomic DNA was digested using *Eco*RI and *Mse*I restriction enzymes (1.25 units/μL); to this end, the following was added: 5 μL of 5 × read buffer [50 mM Tris-HCl (pH 7.5), 50 mM Mg-acetate, 250 mM K-acetate], 12.5 μL of genomic DNA, 2 μL of *Eco*RI and *Mse*I (1.25 units/μL) restriction enzymes and 5.5 μL deionized water. The total volume of the reaction mixture was 25 μL per sample. After adding all the reagents, samples were vortexed and incubated for 3 hours at 37°C and for 15 minutes at 70°C, before being placed on ice. We added 24 μL adapter ligation solution [*Eco*RI/*Mse*I adapters, 0.4 mM ATP, 10 mM Tris-HCl (pH 7.5), 10 mM Mg-acetate, 50 mM K-acetate] and 1 μL T-4 DNA ligase (1 units/μL) to the 25 μL mixture containing the digested DNA. All components were mixed on a vortex and incubated for 3 hours at 20°C. After incubation, the samples were diluted 1:10 using TE buffer [10 mM Tris-HCl (pH 8.0), 0.1 mM EDTA]. The total volume of the diluted samples, after ligation, was 100 μL. At this stage, the samples were frozen at -20°C. In the next stage, preamplification was carried out using 5 μL of the diluted DNA matrix, 40 μL pre-amp primer mix, 3.5 μL 10 × PCR buffer, 1.5 μL MgCl_2_ (0. 25mM), and 1 μL *Taq* polymerase (5 U/μL). The samples were centrifuged and subjected to PCR in a T3 BIOMETRA thermal cycler (Polygen, Gliwice, Poland) set to a program of 27 cycles of denaturation at 94°C for 30 s, primer annealing at 56°C for 60 s, and amplification at 72°C for 60 s. Then, dilution was performed as follows: 3 μL of pre-amplification mixture and 147 μL of TE buffer were added to the sterilized tubes. Samples were frozen at -20°C. Selective amplification was carried out in the next step. We prepared a reaction mixture consisting of 5 μL diluted pre-amplified DNA matrix, 5 μL Mix 1 (*Eco*RI primer 0.18 μl; deionized water 1.22 μL; *Mse*I primer 2 μL; dNTP 1.6 μL) and 10 μL Mix 2 (7.3 μL deionized water; 2 μL 10 × PCR buffer; 0.6 μL MgCl_2_; 0.1 μL *Taq* polymerase). Fifteen specific primer pairs were used to determine the degree of polymorphism between hybrids and their parental components. The samples were placed in a T3 Biometra thermal cycler (Polygen, Gliwice, Poland) set to 2 cycles of denaturation at 94°C for 1 min, primer annealing at 65°C for 1 min, and amplification at 72°C for 2 minutes; followed by 9 cycles with the amplification temperature being lowered by 1°C per cycle; and 35 cycles of denaturation at 94°C for 1 min, primer annealing at 56°C for 1 min, and amplification at 72°C for 2 min. Once the samples were removed from the thermocycler, they were frozen at -20°C. Electrophoresis was performed on a 5% polyacrylamide gel composed of 52.5 g urea; 40 mL deionized water; 6.25 mL TBE buffer (per liter: 10.8 g Tris base; 5.5 g Boric acid; 4 ml 0.5 M EDTA pH 8.0); 15.5 mL 40% acrylamide:bisacrylamide (19:1); 83.75 μL TEMED; 808 μL APS. Electrophoresis was carried out in TBE 0.5 M buffer for 2.5 hours at 2400 V and 400 mA. After electrophoresis, the gel was stained with silver nitrate.

### 2.8 Identification of Random Amplification of Polymorphic DNA Molecular Markers

The PCR analysis of nineteen inbred lines was performed for three biological replicates; it was carried out in 12.5 μl of a mixture composed of 9.75 μL deionized water, 0.125 μL 1M Tris HCl with pH 8.3, 1.0 μL 25 mM MgCl_2_, 0.0625 μL BSA, 0.625 μL2 mM dNTP, 0.25 μL primer at 5 pmol/μL, 0.1875 μL *Taq* polymerase at 5 U/μL, and 0.5 μL extracted DNA at 25 ng/μL. Forty random oligonucleotide primers were used to determine the degree of polymorphism between hybrids and their parental components. The thermal cycler was set to the following program: initial denaturation at 94°C for 1 min; then 10 cycles of denaturation at 94°C for 5 s, primer annealing at 37°C for 30 s, and amplification at 72°C for 30 s; followed by 35 cycles of denaturation at 94°C for 5 s, primer annealing at 37°C for 30 s, and amplification at 72°C for 60 s. After PCR, 1 μL of dye—consisting of 0.25% bromophenol blue, 40% sucrose, and deionized water—was added to each sample. The *Taq* polymerase used in the mixture was purchased from MBI-Fermentas-ABO, whereas the other reagents were purchased from Sigma-Aldrich (Poznań, Poland). Electrophoresis was carried out for 2 h on a 1.5% TBE-agarose gel stained with 1.0 μL ethidium bromide. Electrophoresis was carried out on TBE 1 × buffer at 100 V and 200 mA. To visualize the results, Molecular Imager Gel Doc XR transilluminator and ImageLab Software (Bio-Rad Laboratories, Watford, England) were used.

### 2.9 Statistical Analysis

We compared the genetic diversity of maize hybrids and their parental components using molecular analysis with the GenStat 18 statistical package. The molecular weights of the generated amplification products were recorded in a binary system, with “1” denoting the presence of the product for a given genotype, and “0” denoting its absence. In order to estimate the genetic similarity (GS) of the examined subjects, the following five coefficients were used: Jaccard [23], Kulczyński [24], Sokal and Michener [27], Nei [25], and Rogers [26]. Genetic similarity coefficients ranged from 0 (lack of similarity) to 1 (full similarity).

The genetic similarity values were determined for all tested pairs using the five coefficients. We determined the correlation coefficients between the genetic similarity values using the individual coefficients [29]. The differences were tested for significance at the α = 0.001 level.

The genetic similarity coefficients were used to hierarchically group the objects using the average linkage method.

The relationship between the degree of relatedness and the genetic similarity values calculated using the five coefficients was determined using Pearson’s linear and Spearman’s rank correlation coefficients.

## 3 Results

### 3.1 The Magnitude of the Heterosis Effect

Detailed data on the size of heterosis effects were included in the table presented in Tomkowiak et al. [20], where they were correlated with other molecular markers (SilicoDArT and SNP). Narew and Popis were the most fertile hybrids in the field experiment, showing the greatest significant heterosis effect for most of the yield structure traits in 2012 and 2013 at Łagiewniki. In 2014, the Narew hybrid showed the greatest significant heterosis effect for LC (5.99), LCO (5.32), MKC (111.8), and WTK (146.8), whereas the Popis hybrid showed the greatest effects for NRK (1.93) and yield (9.79). In 2013, the Narew hybrid showed the greatest heterosis effect for DC (0.778), MKC (89.9), and WTK (136.4), whereas the Popis hybrid showed the greatest effect for NRK (2.03), NKR (11.07), and yield (7.72). The results of the field experiment at Smolice in 2013 were similar, with Narew turning out to be the best hybrid. In 2014, Kozak was the best hybrid at Smolice.

### 3.2 Degree of Relatedness

The degree of relatedness between hybrid parental components ranged from 0% to 50% (Table 1). The greatest degree of relatedness was between parental forms of the O Glejt, Wilga, Blask, and Grom hybrids (50%). Parental components of the Popis, Brda, and Kozak hybrids were unrelated (0%).

### 3.3 Genetic Similarity estimated using various molecular marker types

After converting the PCR product sizes of the generated amplification products to the binary system, we obtained 528 AFLP markers, 234 RAPD markers, and 262 SSR markers. Based on the three types of molecular markers used, we constructed the Table 2, showing genetic similarity of the hybrid parental components, using five dissimilarity coefficients in independent analyses. The similarity, as determined by the molecular markers, was correlated with the degree of relatedness of parental forms. Regardless of the coefficient used, the SSR and AFLP molecular markers proved the best for testing the genetic similarity between maize parental components, because of the highest correlation between the genetic dissimilarity indices and the degree of relatedness (Table 1). The similarity determined using the RAPD molecular markers was, for all coefficients calculated, negatively correlated with the degree of relatedness between the parental components. In case of the SSR markers, the highest-scoring coefficient for determining the genetic similarity was the Jaccard coefficient, with the Spearman correlation coefficient of 0.449*. Comparatively, the Rogers coefficient proved to be the best-scoring for the AFLP markers, with a Spearman correlation coefficient of 0.580* (Table 2).

**Table 2:** Correlation coefficients between the heterosis effect in the hybrids and genetic similarity of their parental lines determined based on various types of DNA markers and dissimilarity coefficients

Trait	Selected measures of genetic similarity estimated on the basis of SSR, AFLP and RAPD molecular markers														

	Jaccard [23]			Kulczyński [24]			Nei [25]			Rogers [26]			Sokal and Michener [27]		

	AFLP	RAPD	SSR	AFLP	RAPD	SSR	AFLP	RAPD	SSR	AFLP	RAPD	SSR	AFLP	RAPD	SSR
LC	0.034	0.056	-0.188	0.020	0.051	-0.195	0.018	0.035	-0.201 -	0.619*	-0.507	-0.593*	-0.235	0.327	0.299
DC	0.295	0.143	-0.005	0.285	0.249	-0.017	0.284	0.122	-0.019	-0.326	-0.405	-0.246	-0.111	0.545	0.382
LCO	-0.034	0.093	-0.183	-0.050	0.061	-0.190	-0.052	0.072	-0.196 -	0.614*	-0.540	-0.658*	-0.225	0.376	0.275
DCO	0.475	0.088	0.275	0.474	0.389	0.266	0.473	0.075	0.261	0.062	-0.338	0.005	0.092	0.535	0.444
NRG	0.300	-0.111	-0.035	0.306	0.236	-0.049	0.306	-0.125	-0.044	-0.249	0.143	0.287	-0.065	0.030	0.279
NGR	0.153	-0.084	0.006	0.144	0.337	-0.011	0.143	-0.104	-0.011	-0.469	-0.209	-0.163	-0.009	0.246	0.489
MGC	0.142	0.100	-0.126	0.131	0.133	-0.137	0.131	0.075	-0.141	-0.515	-0.384	-0.440	-0.196	0.318	0.348
WTG	0.082	0.423	-0.356	0.067	-0.304	-0.351	0.065	0.410	-0.359	-0.330	-0.445	-0.604*	-0.219	0.340	-0.185
Yield	0.221	-0.079	-0.095	0.218	0.017	-0.103	0.218	-0.102	-0.106	-0.495	-0.178	-0.213	-0.154	0.070	0.374

LC - cob length, DC - cob diameter, LCO - core length, DCO - core diameter, NRG - number of rows of grain, NGR - number of grain in a row, MGC - mass of grain from the cob, WTG - weight of one thousand grains * P<0.05; ** P<0.01; *** P<0.001

### 3.4 Correlations of Genetic Similarity with the Magnitude of the Heterosis Effect

The genetic similarity between parental components determined using molecular SSR markers (with the Jaccard, Kluczyński, Nei, and Rogers coefficients) proved to correlate negatively with the size of the heterosis effect in the hybrids for all the examined yield structure traits other than DCO and NKR (Table 2). This implies that the greater the genetic distance determined using SSR markers, the greater the heterosis effect for LC, DC, LCO, NRK, MCG, WTK, and Yield. Similar result were found for AFLP molecular markers and the Rogers coefficient: here, the greater the genetic distance between parental components, the greater the heterosis effect in hybrid forms for the LC, DC, LCO, NRK, NKR, MKC, WTK, and Yield traits. Different results were obtained for SSR markers scored with the Sokal and Michener coefficients, because the genetic similarity between the parental components was positively correlated with the size of hybrid heterosis effect for all the crop structure features other than WTK. This implies that the greater the genetic distance between parental components, the smaller the effect of heterosis on the other traits (Table 2).

## 4 Discussion

Carefully selected inbred lines with very good combining ability are needed to obtain high-yielding, high-quality maize hybrids. The breeding success of all plant species, not just maize, is determined by access to starting materials with the greatest possible variety. When choosing the starting material, the breeder must remember that, for multigenetic traits (such as yield), the genetic progress achieved by artificial selection depends mainly on the additive genetic variation—i.e., on the heritability and the genetic variability of the selected feature. For the selection to be effective, the desired traits must be present in the starting germplasm because selection can only alter the frequency of genes. Dividing the starting material into heterotic groups results in an increased efficiency and reduced breeding costs. In the light of those facts, assessment of the combining ability of inbred lines and the selection of testers—as well as the correct interpretation of the results—emerge as some of the most important breeding issues [30].

Combining ability is generally assessed using experimental methods, but statistical and genetic parameters are also used [31]. These parameters include general combining ability (GCA) and specific combining ability (SCA). GCA is the average value of a quantitative trait of hybrid forms obtained by crossing the tested form in numerous combinations. Assessment thus concerns a single parental form being crossed with a number of partners, and is a measure of the average value of one parent’s gametes. The additive effect of genes is fixed and determines the genetic conditioning of the trait. The higher the additive part of variance, the higher the GCA variance [32, 33]. SCA expresses the difference between the predicted overall combining ability and the actual value of a particular cross-combination. It therefore refers to single cross-combinations whose SCA values may be lower or higher than the overall combining value, and is part of the nonadditive potency of genes [34, 35, 36]. The components of nonadditive variance (SCA), dominance, and interaction (epistasis) are highly unstable. The GCA-to-SCA ratio thus offers an opportunity to assess the mode of action of genes that condition a given trait [37, 38].

The best combining abilities (SCA) in our study were recorded between the S64417 and S61328 lines, which were the parental components of the Narew hybrid, and the S54555 and S79757 lines, constituting the components of the Popis hybrid. In 2013 and 2014 at Łagiewniki, both hybrids showed the greatest significant heterosis effect for most of the yield structure traits and for the yield itself. Notably, the parental components of those hybrids are not related to each other (the degree of relatedness of the parental forms of the Narew hybrid is 4%; for the Popis hybrid, this is 0%). The genetic similarity, based on the SSR and the AFLP markers for Jaccard, Kulczyński and Nei measures, between the parental components of the Narew hybrid ranged from 0% to 5%, while for the Popis hybrid this varied from 0% to 14%. It can thus be seen that genetic similarity determined using those markers and measures reflects the degree of relatedness of the parental components.

Yield is an important trait and, at the same time, a very complex one. The crop is influenced by environmental and genetic factors, physiological processes, as well as many other causes. Knowing the level of relationship between traits, their mutual correlations, and the relationship between the parental generation and its offspring, can help properly select the lines for crossing, thereby allowing an increase in yield [39]. The analysis of the relationship between the yield and the yield-forming traits, especially the yield components, thus makes it possible to describe the conditioning of the yield by the respective plant traits, while providing knowledge about their quantitative role in shaping the yield. The mechanism of plant yield and the selection criterion for yield in a breeding program are therefore very important subjects of research [40, 41].

In this study, Narew, Popis, and Kozak were the most fertile hybrids. Both the Narew and Popis hybrids showed the greatest significant heterosis effect for most of the yield structure traits in 2013 and 2014 at Łagiewniki. At Smolice in 2013, Narew was also the hybrid with the greatest heterosis effect for LC, DC, NRK, MKC, and WTK, whereas in 2014 Kozak turned out the best hybrid. Parental components of these hybrids may serve as valuable material for heterosis crosses.

The first attempts to correlate the estimated genetic similarity between parental forms and the heterosis effect were made as early as 1966 [42]. In recent years, many researchers have also attempted to infer the effect of heterosis by studying the genetic distance between parental lines [43, 44]. The phenomenon of heterosis has been looked into many times, including by Girke et al. [45], and Jiang et al. [46]. The literature indicates that heterosis cannot be explained by any single hypothesis, and that the causes of heterosis depend on species, traits, and parental combinations [47]. Becker and Link [48], who examined the relationship between the genetic distance of inbred maize lines and the heterosis effect, found that dent × dent maize hybrids derived from parental forms with a large genetic distance gave the greatest heterosis effect on grain yield. In a recent study of maize, Frisch et al. [49] showed that prediction of hybrid performance with transcriptome-based distances is very precise.

Many researchers have discussed the relationship between genetic similarity and heterosis. In 2003, Betrán et al. [50] showed a positive correlation between the heterosis effect in maize in seed yield with genetic similarity in parental forms. Riaz et al. [51] also pointed to the usefulness of molecular markers for selecting parent components for heterosis crossing in their study of *Brassica*. In 2007, Liersch and Bartkowiak [52] showed a relationship between genetic similarity and heterosis in *Brassica*. Tomkowiak et al. [20] in maize and Plieske and Struss in *Brassica* [53] came to similar conclusions. Different results were shown by Teklewold and Becker [54] for Abyssinian mustard and Yu et al. [55] for *Brassica*. These authors emphasized that genetic similarity derived from molecular markers is not sufficient to predict the effects of heterosis. Radoev et al. [56] and Kramer et al. [57] believe that heterosis can be predicted using a marker coupled to the QTL of interest to the grower. Lariepe et al. [58] noticed that many alleged overdominant Quantitative Trait Loci could correspond to pseudo-overdominance, where two (or more) linked dominant QTLs are in repulsion.

The lack of unambiguous relationships between the genetic distance determined using RAPD markers and the heterosis effect may be due to the fact that this method has the weakness of generating dominant markers and requires preselection of a primer that gives stable and clear electropherograms. The amplification reaction of RAPD markers was found to be highly sensitive to changes in the concentration and source of Taq polymerase, fluctuations in the amount of plant tissue extract, and the magnesium and potassium cation content. Many authors have also pointed out that the greater the genetic dissimilarity of parental lines, the greater the effect of heterosis and that as a result of crossing genetically differentiated lines with greater frequency, the fertile hybrids are obtained [57, 59, 60, 61]. We can thus conclude based on our research that SSR molecular markers (for the Jaccard, Kluczyński, Nei, and Rogers coefficients) are the best for preselection of the parental components for heterosis crossing.

## 5 Conclusions

We still do not know much about the phenomenon of heterosis and its mechanisms, and can only utilize the vigor of F_1_ hybrids to create heterosis varieties. This is an important problem for the methodology of plant breeding. It is expected that breeding progress can be obtained through studies of the nuclear genomes and of plasmons and their interdependence and interaction with the environment. Our analysis has demonstrated that the initial selection of parental components for heterosis crossing using molecular markers can be carried out using the SSR technique with the Jaccard, Kluczyński, Nei, and Rogers coefficients, as the high polymorphism of SSR markers allows to distinguish the closely related individuals. Molecular AFLP markers have proved less useful in selecting parental components for heterosis crossing, but they may be used to group the lines with incomplete origin data. All this demonstrates the necessity of using other techniques, such as SSR, to select genotypes for heterosis crosses.
